# The Clinical Manifestations, Risk Factors, Etiologies, and Outcomes of Adult Patients with Infectious Meningitis and Encephalitis: Single Center Experience

**DOI:** 10.3390/neurolint16050073

**Published:** 2024-09-05

**Authors:** Seraj Makkawi, Shatha Alqurashi, Wejdan Hubayni, Saleha Almahdawi, Sadeem Bahkali, Abeer Alharbi, Osama Khojah, Aisha Halawani, Israa Malli

**Affiliations:** 1 College of Medicine, King Saud bin Abdulaziz University for Health Sciences, Jeddah 22384, Saudi Arabiahubayni217@ksau-hs.edu.sa (W.H.);; 2 King Abdullah International Medical Research Center, Jeddah 22384, Saudi Arabia; halawaniai@mngha.med.sa; 3 Department of Neuroscience, Ministry of the National Guard-Health Affairs, Jeddah 22384, Saudi Arabia; 4 Department of Medical Imaging, Ministry of the National Guard-Health Affairs, Jeddah 22384, Saudi Arabia

**Keywords:** meningitis, encephalitis, bacterial meningitis, viral meningitis, viral encephalitis, central nervous system infection

## Abstract

(1) Background: Central nervous system (CNS) infections, including meningitis and encephalitis, are serious conditions which are associated with high morbidity and mortality. This study aims to identify the clinical manifestations, etiologies, and outcomes of meningitis and encephalitis in adult patients in Saudi Arabia, addressing the current gap in understanding these conditions within this population. (2) Methods: This is a single-center retrospective study which included all adult patients diagnosed with meningitis and encephalitis from March 2016 to May 2022. (3) Results: This study found that most cases of meningitis and encephalitis occurred due to unknown pathogens. Pretreatment with antibiotics prior to lumbar puncture (LP) was found in 71.2% of patients with meningitis. Altered mental status and seizures were common presenting symptoms among patients with encephalitis while altered mental status and fever were common among patients with meningitis. (4) Conclusions: Adherence to guidelines in treating meningitis and encephalitis and performing LPs in a timely manner are important. Establishing national biobanks with biological samples from patients suspected of having meningitis or encephalitis will significantly enhance our understanding of these conditions in Saudi Arabia.

## 1. Introduction

Central nervous system (CNS) infections encompass a range of conditions affecting the nervous system, including meningitis and encephalitis, both of which can be potentially severe [1]. Meningitis involves inflammation of the meninges, the protective membranes covering the brain and spinal cord, while encephalitis is characterized by inflammation of the brain tissue itself. These infections can be caused by a variety of pathogens, including bacteria, viruses, fungi, and parasites, making diagnosis and treatment complex and challenging [2]. Meningitis and encephalitis are neurological emergencies which require quick diagnosis and timely initiation of appropriate treatment [3]. Meningitis and encephalitis can present with a variety of clinical manifestations, depending on the patient’s age and immune status [4]. Common symptoms of meningitis include fever, neck stiffness, and headache, whereas encephalitis typically presents with altered mental status, fever, and seizures [4]. Vaccinations against *Haemophilus influenzae type B*, Streptococcus pneumoniae, *Neisseria meningitidis*, and varicella are standard for all newborns and children in Saudi Arabia [5,6]. For national and international pilgrims, vaccination against *Neisseria meningitidis* is mandatory [6]. The etiology of meningitis and encephalitis is often undetermined [7]. Bacterial meningitis is primarily caused by Streptococcus pneumoniae and Neisseria meningitidis, but their incidences have drastically decreased due to the introduction of vaccines [4,7]. In cases of viral meningitis, a study in Saudi Arabia found enterovirus to be the most common causative agent of aseptic meningitis [6]. Viruses are the most common causative agents of encephalitis, with viral agents varying by geographical area [4,8,9]. However, data on the etiology of encephalitis in the adult population in Saudi Arabia is not available. Previous studies of meningitis and encephalitis in Saudi Arabia were almost exclusively represented by the childhood age group. Therefore, there is a paucity of research discussing meningitis and encephalitis in the adult population in Saudi Arabia. To fill this literature gap, the purpose of this study is to identify the clinical manifestations, etiologies, and outcomes of infectious meningitis and encephalitis in adult patients in Saudi Arabia. Identifications of these measures could help in gaining a better understanding of how the disease affects the adult population in Saudi Arabia.

## 2. Materials and Methods

This was a retrospective cohort study where the patients were included using a non-probability consecutive sampling technique to select participants. All patients diagnosed with meningitis or encephalitis at King Abdulaziz Medical City, Jeddah, Saudi Arabia, between March 2016 and May 2022 were reviewed. The ICD-10 codes G00 “Bacterial meningitis, not elsewhere classified”, G01 “Meningitis in bacterial diseases classified elsewhere”, G02 “Meningitis in other infectious and parasitic diseases classified elsewhere”, G03 “Meningitis, unspecified”, and G04 “Encephalitis, myelitis and encephalomyelitis” were used to retrieve and review cases. Diagnosis was confirmed by independently assessing all adult patients (≥18 years) for CSF analysis showing white blood cells with ≥5 cells/mm^3^ and/or identification of a pathogen through CSF culture or FilmArray^®^ Meningitis Encephalitis Panel Testing. The panel, used in our institution, uses CSF specimens to identify the following pathogens: *Escherichia coli K1, Haemophilus influenzae*, *Listeria monocytogenes*, *Neisseria meningitidis (encapsulated)*, *Streptococcus agalactiae*, *Streptococcus pneumoniae*, cytomegalovirus, enterovirus, herpes simplex virus 1, herpes simplex virus 2, human herpesvirus 6, human parechovirus, varicella zoster virus, and cryptococcus neoformans/gattii. Further testing for other pathogens is performed on a case-by-case basis. Clinical features and brain MRI findings were relied upon to reach the diagnosis in two of our patients because there was a contraindication to a lumbar puncture (LP) or patients did not consent to the LP. Classification of causative pathogens was based on meningitis/encephalitis panel or CSF culture. Data collected from the patients’ electronic medical records included: demographic data, comorbid diseases, clinical manifestations at presentation (level of consciousness, temperature, headache, photophobia, neck stiffness, nausea, vomiting, and/or focal neurological deficits), CSF analysis (white blood cells count, red blood cells count, glucose level, total protein level, meningitis/encephalitis panel, and culture), neuroimaging results, and outcomes. Patients who returned to their baseline functional status were deemed to have a favorable outcome. Data analysis was conducted using JMP software (John’s Macintosh Project), version 15.0 (SAS Institute Inc., Cary, NC, USA). The means and standard deviation were used for continuous variables that had a normal distribution. Continuous variables that were not normally distributed are reported using the median and interquartile range. Ethical approval was obtained from King Abdullah International Medical Research Centre’s Institutional Review Board (IRB) on 13 March 2022.

## 3. Results

### 3.1. Demographics and Clinical Features

The study included 50 patients diagnosed with meningitis or encephalitis, ranging in age from 18 to 79 years with a gender predilection to males (M:F 3.1:1). Meningitis accounted for 72% of our cohort. The most common risk factors seen in patients with meningitis included diabetes mellitus and recent neurosurgical operation. Diabetes mellitus was also the most common risk factor for encephalitis cases, but none of them had a history of recent neurosurgery or a ventriculoperitoneal shunt placement. Patients with meningitis commonly presented with altered mental status, followed by headache, fever, and vomiting. Only 5.6% had the classic triad of fever, neck stiffness, and an altered mental status, but 72.2% had two out of the four symptoms of headache, fever, neck stiffness, and an altered mental status. Seizure was the most frequent presentation observed in patients with encephalitis. The demographics and clinical features are displayed in Table 1.

### 3.2. Etiology

The underlying cause of meningitis was determined in a third of cases. Among those cases with identifiable causes, *Neisseria meningitidis* and *Klebsiella pneumoniae* (5.6%) emerged as the prevailing pathogens responsible for the condition. All but one of the meningitis cases with a known pathogen were due to bacterial infections, with 63.6% of these bacteria being Gram-negative organisms. In encephalitis cases, the etiology behind half of the cases was not identified. Among cases identified as associated with a viral organism, Herpes simplex virus-1 (HSV-1) accounted for around a third of the cases. Table 2 shows the causative organisms behind meningitis and encephalitis.

### 3.3. Investigations

**Figure 1:**
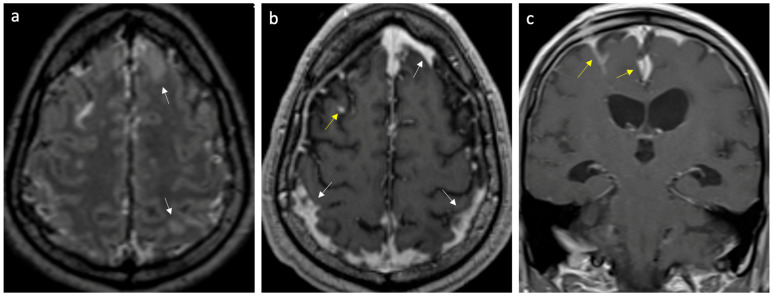
Case of meningitis secondary to *Klebsiella pneumoniae*. (**a**) Axial FLAIR post-gadolinium image reveals enhancing leptomeninges over the frontoparietal regions and scattered parenchymal edema (arrows). (**b**,**c**) Axial and coronal T1 post-gadolinium images show thick, intense leptomeningeal enhancement (yellow arrows) and pachymeningeal enhancement (white arrows).

**Figure 2:**
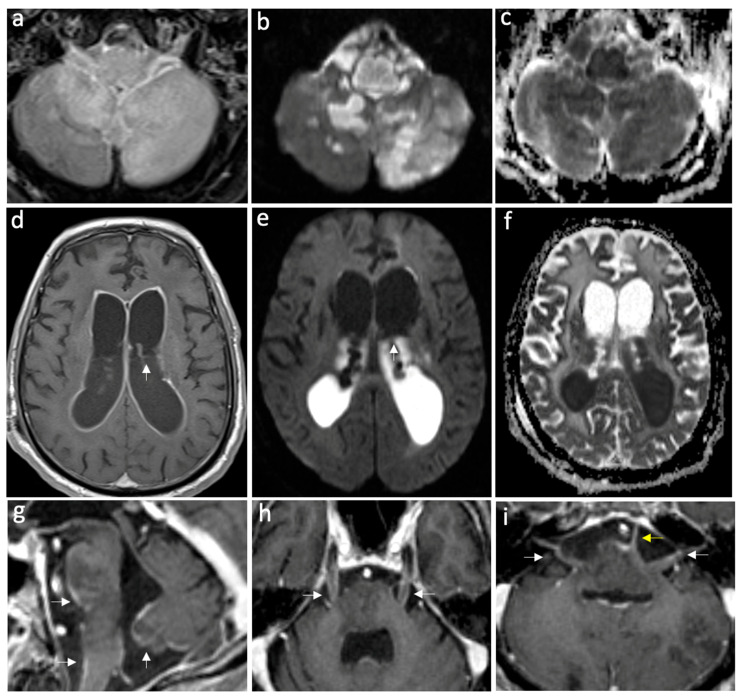
Case of complicated meningoencephalitis with ventriculitis and empyema secondary to *Salmonella enterica*. Axial FLAIR post-gad (**a**), DWI, and ADC map (**b**,**c**) show abnormal parenchymal hyperintensities and diffusion restriction in the medulla oblongata and bilateral cerebellar hemispheres, consistent with encephalitis. Furthermore, note the restricted diffusion in the prepontine cistern, consistent with empyema. Axial T1 post-gadolinium (**d**), DWI, and ADC map (**e**,**f**) show intense ventricular ependymal surface enhancement related to ventriculitis and intraventricular diffusion restriction due to intra-ventricular empyema, with a pus fluid level indicated by white arrows. On the sagittal post-gadolinium image (**g**), leptomeningeal enhancement is visible on the ventral pons, medulla oblongata, and inferior vermis, indicating meningitis (white arrows). The axial post-gadolinium images show leptomeningeal enhancement along the bilateral trigeminal nerves (**h**), bilateral facial and vestibulocochlear nerves (white arrows), and the left abducent nerve (yellow arrow) (**i**).

In cases of meningitis, the median CSF WBC count was 235 leukocytes/µL, mostly polymorphonuclear (65.4%), and the median CSF RBC count was 20 cells/µL. The median levels of CSF protein and glucose were 1.13 mg/mL and 3.5 mmol/L, respectively. Table 3 shows the results of the CSF analyses in patients with encephalitis and meningitis. Tables S1 and S2 show the individual results of the CSF analyses in patients with meningitis and encephalitis, respectively. Brain computed tomography (CT) was performed on 88.9% of these patients, of whom 28.12% showed one or more abnormality. Brain magnetic resonance imaging (MRI) was performed on 58.83% of patients, of whom 66.7% had an abnormality. Of those who underwent neuroimaging, an associated hydrocephalus or ventriculitis was seen in 15.63% cases, and an associated abscess was seen in 6% of cases. In the following figures, brain MRI images are presented illustrating two cases: one of a patient diagnosed with meningitis (Figure 1) and another with meningoencephalitis (Figure 2).

In patients with encephalitis, the median CSF WBC count was 14 leukocytes/µL and the median CSF RBC count was 4 cells/µL. The median levels of CSF protein and glucose were 0.66 mg/mL and 4.95 mmol/L, respectively. Brain CT was performed in 92.85% of patients, and only one case exhibited an abnormal CT. The abnormal CT imaging showed hypodensity in the right insula and temporal pole. All patients underwent brain MRI, 85.71% of whom had abnormal imaging. The most frequently observed abnormalities were temporal lobe changes such as cortical and subcortical increased T2 signal. Of those who underwent neuroimaging, ventriculitis was observed in one of the patients.

### 3.4. Management and Outcomes

In patients with meningitis, antimicrobial therapy was given in all patients, a combination of antibiotics and antiviral in 66.7%, antibiotics alone in 25%, and antiviral alone was given in one case (2.8%). Adjunctive corticosteroids within 72 h were used in (69.4%) of patients. Notably, antimicrobial therapy was initiated prior to performing an LP in 71.2% of patients. In patients with encephalitis, all cases were started on antiviral therapy, and a combination of antibiotics and antiviral was given in 92.85% of patients. Antiviral therapy was continued in patients with evidence of HSV infection. Adjunctive corticosteroids within 72 h were used in half of the encephalitis cases. Antimicrobial therapies were later adjusted based on the results of the culture and sensitivity.

In cases of meningitis, 22.2% of patients were admitted to ICU for a median of 24 days. On the other hand, more than two-thirds of patients with encephalitis were admitted to ICU (71.42%) for a median of 18 days. Only 8.3% of meningitis patients were admitted for 90 days or more, with a median length of stay of 14 days. Upon discharge, most patients with meningitis were back to their baseline (86.1%), and 33.3% of them were discharged on one or more antiseizure medications. In cases of encephalitis, 64.3% returned to their baseline, and 64.3% were discharged on one or more antiseizure medications. Only 14.3% of encephalitis patients were admitted for 90 days or more, with a median length of stay of 23.5 days. In-hospital mortality rates in patients with meningitis and encephalitis were 5.6% and 35.7%, respectively.

## 4. Discussion

In this study, we investigated the clinical manifestations, etiologies, and outcomes in adult encephalitis and meningitis patients in a single large medical center in Saudi Arabia. Altered mental status was the most common clinical presentation among the meningitis patients, while seizure was the most common clinical presentation among the encephalitis patients. We found that two-thirds of the meningitis cases and about half of the encephalitis cases were attributed to unidentified pathogens. However, the most common known causes of meningitis were *Neisseria meningitidis* and *Klebsiella pneumoniae*, while HSV-1 was the most common known cause of encephalitis. In-hospital mortality was 35.7% among patients with encephalitis, while it was 5.6% in patients with meningitis.

There are no large studies on the prevalence of meningitis or encephalitis in adults in Saudi Arabia to fully understand the burden, prevalence, or etiology. However, a single-center study indicated that adults represent 37% of bacterial meningitis cases and 15.4% of aseptic meningitis cases [8,9]. Alhazmi et al. conducted a retrospective study on bacterial meningitis in Jazan, Saudi Arabia, identifying *Pseudomonas aeruginosa* as the most common causative organism [9]. This was likely due to the high prevalence (92%) of hospital-acquired meningitis [9]. Pathogens have been increasingly showing resistance to common antimicrobials. Alhazmi et al. reported that 12.2% of hospitalized patients with meningitis in a tertiary hospital in Saudi Arabia had an extended-spectrum beta-lactamase (ESBL) pathogen [9]. Peng et al. reported high rates of resistance in coagulase-negative *Staphylococcus* (72.3–80.5%), ESBL-producing *E. coli* (44.4–49.2%), and *K. pneumoniae* (55.6–88.9%) among pediatrics with bacterial meningitis at 13 children’s hospitals in China [10]. This poses a major challenge in treating patients with bacterial meningitis and emphasizes the need for the judicious use of empiric antibiotics. Our study, which also included viral meningitis, found that 91.6% of cases with an identifiable organism were related to bacterial infections, with *Neisseria meningitidis* and *Klebsiella pneumoniae* being the most commonly identified organisms. This finding aligns with the literature identifying *Neisseria meningitidis* as one of the common causes of bacterial meningitis [11]. Aldriweesh et al. conducted a retrospective study on aseptic meningitis in Riyadh, Saudi Arabia, identifying enterovirus and varicella zoster virus (VZV) as the most common pathogens in adults followed by HSV-2, cytomegalovirus, and human herpesvirus 6 [8]. Similarly, cases were commonly linked to *enterovirus* and *human herpesvirus* 6 infections in patients younger than 18 years [8]. Common causes behind infectious encephalitis seem to differ geographically [7]. Ben Abid et al. conducted a retrospective study in Qatar on CNS infections caused by viral pathogens by analyzing CSF samples and identified Epstein–Barr virus as the most common cause of viral encephalitis [7]. Two studies on adult patients with encephalitis, conducted in Switzerland, France, and the UK, found HSV-1 to be the predominant organism [12,13,14]. Creating national biobanks with biological samples from patients suspected of CNS infections will substantially advance our understanding of these conditions in Saudi Arabia.

Performing an urgent LP in patients with a suspected CNS infection is crucial to determine the etiology and to guide treatment and disposition [15]. Although the LP should ideally be performed within one hour of hospital arrival, this recommendation is seldom followed [15]. Administering antimicrobial therapy before extracting the CSF sample can affect the results of the Gram stain and culture, potentially leading to unidentifiable organisms [16]. However, this approach does not seem to affect CSF cell counts and protein levels [16]. Rogers et al. reported that 66% of patients with healthcare-associated ventriculitis and meningitis underwent LP after receiving antibiotics and found that this significantly affected the results of Gram stain and culture [16]. Consequently, nearly half of their cohort had a negative CSF culture [16]. Alhazmi et al. evaluated CSF samples to determine the organisms behind bacterial meningitis and found high variability in positivity rates across the study period, hypothesizing that antibiotic pretreatment was a contributing factor [9]. Similarly, our study found that 71.2% of meningitis cases received antibiotics prior to undergoing the LP. This contributed to having two-thirds of the meningitis cases and about half of the encephalitis cases without a clear organism known. This finding is worrying because it affects the ability to accurately diagnose and treat these infections, potentially leading to suboptimal patient outcomes and complicating epidemiological tracking and management of infectious diseases. With all of that being said, it is also imperative not to delay antimicrobial treatment. It has been shown that delaying antibiotics is linked to in-hospital mortality and poor outcomes [3]. Therefore, a balanced approach that prioritizes timely antimicrobial therapy while minimizing delays in LP is crucial for optimal patient care and effective management of meningitis. The challenge with treating an unknown pathogen causing meningitis or encephalitis is not exclusive to bacteria. Multiple studies conducted in Saudi Arabia, Qatar, the USA, and the UK have reported aseptic meningitis without a known causative pathogen, with rates ranging from 42% to 81% showing that even with advancements in diagnostic methods, diagnosing viral CNS infections remains difficult [7,8,17,18]. Metagenomic next-generation sequencing (mNGS) is an innovative and transformative approach to diagnosing infectious meningitis and encephalitis. It has facilitated the identification of bacterial, viral, parasitic, and fungal causes of meningitis and encephalitis that had previously eluded diagnosis through standard methods [19]. mNGS is potentially unaffected by administration of antimicrobial therapy before specimen collection [20,21]. Ultimately, a comprehensive understanding of the factors contributing to delays in LPs and strategies to address them, of the impact of treating infectious meningitis or encephalitis caused by an unknown pathogen which includes functional outcomes, morbidity, mortality and length of stay, and of the utility of mNGS in the diagnosis of infectious meningitis and encephalitis are needed.

The most common symptoms among patients with meningitis in our study were altered mental status (69.4%) followed by headache (63.9%) and by fever (58.8%).Two out of the four symptoms (headache, fever, neck stiffness, and an altered mental status) were seen in 72.2% of meningitis patients as opposed to the classic triad that was rarely found in our cohort (5.6%). Previously, van de Beek et al. showed that looking for two symptoms out of the four has a better yield than looking for the classic triad (95% vs. 44%) [22]. They also found that the triad is more likely to appear in meningitis secondary to *Streptococcus pneumoniae* [22]. Aldriweesh et al. found fever, headache, and vomiting to be the most common symptoms in aseptic meningitis [6]. In patients with encephalitis, we found that most patients presented with a seizure or an altered mental status. Mailles et al., who conducted a retrospective study on infectious encephalitis in France, found that most patients presented with an altered mental status (95%) and only a third with a seizure [13]. Granerod et al. included infectious and non-infectious encephalitis and found that fever, headache, seizures, and lethargy affected more than 50% [14]. They also found that symptoms may differ based on the etiology [14]. Fever was found in patients with infectious causes except for VZV encephalitis [14]. Seizures in encephalitis were also shown to be related to etiology with a significant association with HSV encephalitis [23].

Our study demonstrated a low mortality rate among meningitis patients (5.6%). Akaishi et al. conducted a nationwide retrospective study in Japan and found that among 10,338 patients with bacterial meningitis, the mortality rate was 9.5%, significantly associated with *Staphylococcus aureus* [24]. A Dutch study found that the mortality rate among patients with bacterial meningitis was 21%, predominantly due to pneumococcal meningitis [22]. Akaishi et al. also reported that the mortality rate among patients with viral meningitis was 0.61%, significantly associated with HSV [24]. These findings highlight the variability in mortality rates across different regions and underscore the importance of localized studies to better understand and address the factors influencing patient outcomes. Our study demonstrated a high mortality rate among encephalitis patients (35.7%), which surpasses that previously reported in the literature. Out of the five patients with encephalitis who died, two of them were diagnosed with HSV encephalitis, one with CMV encephalitis, and two were without a known pathogen. A large prospective study in the UK showed a mortality rate of 12% among encephalitis patients (including non-infectious causes), mostly attributed to *Mycobacterium tuberculosis* and VZV [14]. Similarly, Mailles et al. showed a mortality rate of 10% [13]. The ENCEIF study showed that almost half of the mortality rate (8%) occurred in patients with HSV encephalitis or VZV encephalitis [25]. This large discrepancy is likely due to the small sample size in our cohort which was largely represented by HSV encephalitis and VZV encephalitis. Additionally, our institution is a tertiary care center with a well-equipped intensive care unit, where severe cases might be referred, which may have artificially increased the mortality rate.

Predilection to males has been previously reported by Aldriweesh et al. who studied aseptic meningitis in Riyadh, Saudi Arabia (54.6%), and Ben Abid et al. who studied CNS viral infections in Qatar (65%) [7,8]. This predilection was also described in China (66% of encephalitis and 69% of meningitis cases), Vietnam (65% of viral CNS infections), Spain (69.8% of viral meningitis cases), and France (53% of aseptic meningitis cases) [26,27,28,29]. The gender differences in our study could be due to male gender predominance in the geographic area. According the Saudi General Authority for Statistics, the ratio of male to female is about 1.56 in the city of Jeddah [30].

### Limitations

This study has several potential limitations, which include its single center retrospective nature and the small sample size which may limit generalizability. Chart review studies have inherent selection bias and potential for missing data, because they depend on existing records that may be incomplete. Most patients received pretreatment of antimicrobials which hindered the ability to identify the possible causative pathogen. Viral identification relied mainly on panel testing but did not utilize advanced techniques such as mNGS. Antimicrobial resistance and its association with morbidity and mortality were not studied which might have been pertinent to explaining the mortality rates. Patients with an acute and severe presentation, such as sepsis, might not have undergone an LP, potentially impacting the reported mortality rate. Finally, this study did not take into account non-infectious etiologies of meningitis or encephalitis.

## 5. Conclusions

Establishing national biobanks with biological samples obtained from patients suspected of having meningitis or encephalitis will significantly enhance our understanding of these pathologies in Saudi Arabia. The majority of meningitis and encephalitis cases were due to unknown pathogens, with most meningitis patients receiving antibiotics prior to LP. It is important to adhere to the guidelines and to perform LP in a timely manner.

## Figures and Tables

**Table 1:** Demographics and clinical characteristics of patients with meningitis and encephalitis.

Variables	Encephalitis n (%) n = 14	Meningitis n (%) n = 36
Male gender	11 (78.6%)	23 (63.9%)
Median age (IQR)	59 (33.3)	47 (33)
**Comorbidities and risk factors**		
Diabetes mellitus	5 (35.7%)	14 (38.9%)
Use of Immunosuppressive drugs	2 (14.3%)	6 (16.7%)
Autoimmune disease	2 (14.3%)	2 (5.6%)
Recent neurosurgery	0	7 (19.4%)
Ventriculoperitoneal shunt	0	4 (11.1%)
Human immunodeficiency virus	0	1 (2.8%)
**Signs and symptoms**		
Seizure	13 (92.9%)	8 (22.2%)
Altered mental status	12 (85.7%)	25 (69.4%)
Fever	6 (42.9%)	21 (58.8%)
Headache	3 (21.4%)	23 (63.9%)
Nausea/Vomiting	3 (21.4%)	19 (52.8%)
Photophobia	0	15 (41.7%)
Neck stiffness	0	9 (25%)
**Outcomes**		
Median Length of Stay (days)	23.5 (17)	14 (15)
Intensive care unit (ICU) admissions	10 (71.4%)	9 (25%)
In-hospital mortality	5 (35.7%)	2 (5.6%)

**Table 2:** The etiology of encephalitis and meningitis.

Variables	Encephalitis n (%) n = 14	Meningitis n (%) n = 36
Herpes simplex virus 1	5 (35.7%)	0
Varicella zoster virus	2 (14.3%)	1 (2.7%)
*Neisseria meningitidis*	0	2 (5.6%)
*Klebsiella pneumoniae*	0	2 (5.6%)
*Acinetobacter baumannii*	0	1 (2.8%)
*Bacillus megaterium*	0	1 (2.8%)
Brucella spp.	0	1 (2.8%)
Enterovirus	0	1 (2.8%)
*Mycobacterium tuberculosis*	0	1 (2.8%)
*Salmonella enterica*	1 (2.8%)	1 (2.8%)
*Streptococcus gordonii*	0	1 (2.8%)
*Streptococcus intermedius*	0	1 (2.8%)
*Streptococcus pneumoniae*	0	1 (2.8%)
Unknown	6 (42.9%)	22 (61.1%)

**Table 3:** The results of CSF analysis.

Variables	Encephalitis n (%) n = 14	Meningitis n (%) n = 36
Median CSF WBC * (IQR)	14 (100)	235 (449)
Median CSF RBC ** (IQR)	4 (10)	20 (124.5)
Median CSF glucose *** (IQR)	4.95 (2.5)	3.5 (2.1)
Median CSF protein **** (IQR)	0.66 (0.4)	1.13 (1.5)

* leukocytes/µL ** cells/µL *** mmol/L **** mg/mL. Abbreviations: CSF: cerebrospinal fluid; WBC: white blood cell; RBC: red blood cell.

## Data Availability

The raw data supporting the conclusions of this article will be made available by the authors on request.

## Supplementary Materials

The following supporting information can be downloaded at: https://www.mdpi.com/article/10.3390/neurolint16050073/s1, Table S1: Individual results of cerebrospinal fluid analysis for meningitis patients. Table S2: Individual results of cerebrospinal fluid analysis for encephalitis patients.
