# Supplementary material for: The Clinical Manifestations, Risk Factors, Etiologies, and Outcomes of Adult Patients with Infectious Meningitis and Encephalitis: Single Center Experience
Source: Neurol Int. 2024 Sep 5;16(5):966–75. doi: 10.3390/neurolint16050073 (PMC11417761; doi:10.3390/neurolint16050073)
Supplement: Supplementary file 1 [file neurolint-16-00073-s001.zip › neurolint-3090743-supplementary.pdf]

Table S1: Individual results of cerebrospinal fluid analysis for meningitis and encephalitis patients

Patient Number	RBC cells/µL	WBC leukocytes/µL	Glucose mmol/L	Protein mg/mL
M1	10	2336	3.9	0.98
M2	44	372	2.6	1.82
M3	20	290	3	1.06
M4	8	34	2.9	0.46
M5	57	480	3.1	0.81
M6	0	1667	4.6	7.09
M7	58	18	4.4	0.91
M8	160	8	3.5	1.92
M9	278	280	1.4	4.17
M10	8	10	6.4	0.66
M11	144	200	5.4	2.08
M12	8	60	3.8	0.3
M13	8	1350	2.3	2.02
M14	0	96	6.2	2.86
M15	1120	1760	3.9	3.39
M16	20	60	3.9	0.62
M17	8	10	6.4	0.66
M18	1020	1980	0.3	0.31
M19	10	8	6.4	0.66
M20	10	44	2.2	2.33
M21	0	10	4.8	0.52
M22	130	390	0.3	1.74
M23	32	292	3.2	1.13
M24	30	270	2.6	0.66
M25	20	510	1	2.81
M26	10	110	5	0.69
M27	220	468	0.4	1.51
M28	0	8	3.6	0.26
M29	2	40	4.9	1.09
M30	800	1240	0.3	11.6
M31	4	90	2.8	NA
M32	20	1290	2.6	1.06
M33	10	50	5.2	1.87
M34	>10	>10	3.9	3.39
M35	140	480	1.3	1.15
M36	500	940	3.5	3.86

Table S2: Individual results of cerebrospinal fluid analysis for encephalitis patients

Patient Number	RBC<br>cells/µL	WBC<br>leukocytes/µL	Glucose<br>mmol/L	Protein<br>mg/mL
E1	0	8	6.6	0.33
E2	360	140	2.7	0.5
E3	0	120	4.1	0.42
E4*	NA	NA	NA	NA
E5	4	34	5.6	0.65
E6	10	18	4.7	0.71
E7	4	8	9.4	0.89
E8	10	290	4.2	1.17
E9	6	8	4.5	0.38
E10	0	104	6.8	0.29
E11	60	8	5.2	4.41
E12*	NA	NA	NA	NA
E13	0	10	2.4	0.66
E14	0	8	10.4	0.77

* Lumbar puncture was contraindicated in these patients, diagnosis was made based on clinical features and neuroimaging findings.
